# Development of a stable low-copy mini F plasmid derivative for evaluating β-lactamase substrate specificity through antimicrobial susceptibility testing

**DOI:** 10.1128/spectrum.03360-25

**Published:** 2026-04-16

**Authors:** Haruka Nakagawa Kamura, Kotaro Aoki, Kohji Komori, Kazuhiro Tateda, Yoshikazu Ishii

**Affiliations:** 1 Center for the Planetary Health and Innovation Science (PHIS), Hiroshima University, The IDEC Institute, Higashi-Hiroshima, Japan; 2 Department of Microbiology and Infectious Diseases, Toho University School of Medicine, Tokyo, Japan; 3 Division of Collaborative Regional Infection Control, Department of Community Well-being, Toho University School of Medicine, Tokyo, Japan; Universita degli Studi dell'Insubria, Varese, Italy

**Keywords:** β-lactamase, β-lactamase substrate specificity, antimicrobial susceptibility testing, mini F plasmid derivative

## Abstract

Traditionally, the impact of the substrate specificity of β-lactamase produced by bacteria on β-lactam resistance (β-lactamase-mediated resistance) has been assessed by performing antimicrobial susceptibility testing (AST) on transformants carrying the cloned gene on arbitrarily chosen β-lactamase-free plasmid vectors. However, because the promoter strength and plasmid copy number of these vectors are not standardized, the resulting β-lactamase expression levels vary, further hampering cross-study comparisons. We constructed pMiniF-1 by inserting a *tac* promoter, a ribosome-binding site, and transcriptional terminators into the mini F plasmid, which is maintained at one to two copies per cell. This design enables controlled β-lactamase expression for accurate evaluation of β-lactamase-mediated resistance via AST. This approach enables the determination and comparison of MICs with β-lactamase expression levels controlled to be as nearly identical as possible. We performed AST on *Escherichia coli* DH5αΔ*bla*_EC-like_ transformants expressing 22 β-lactamase genes (Ambler classes A–D), including carbapenemases that were cloned into pMiniF-1. The resulting antimicrobial susceptibility profiles and MIC differences did not contradict the known substrate specificities of the enzymes. Meropenem and imipenem MICs for the IMP-6 producer were fourfold higher and eightfold lower, respectively, than IMP-1. Aztreonam MICs were similar between transformants lacking β-lactamase genes and those with class B β-lactamases. Imipenem MIC for the OXA-48 producer was ≥8-fold higher than for OXA-1, whereas cefepime MICs were 16-fold lower. We demonstrate that pMiniF-1 enables the comparison of β-lactamase substrate specificity via AST. The pMiniF-1 plasmid (Catalog No. RDB21122) is available from the RIKEN BioResource Research Center (Tsukuba, Ibaraki, Japan) (https://web.brc.riken.jp/en/).

**IMPORTANCE:**

β-lactamases play a central role in bacterial resistance to β-lactam antibiotics, and novel variants continue to emerge. However, the impact of individual β-lactamase variants on β-lactam resistance across diverse enzyme-substrate combinations remains insufficiently characterized. Traditional kinetic analyses require enzyme purification and multiple reaction assays, which hinder comprehensive experimental evaluation. Expression-based approaches have also been used to assess β-lactamase function, but conventional systems depend on unstable high-copy plasmids, raising concerns about reproducibility due to variable plasmid copy numbers. In this study, we developed pMiniF-1, a stable, low-copy plasmid that enables consistent gene expression without antibiotic selection, thereby maintaining stable copy numbers under drug-free conditions. Using 22 β-lactamase genes, we demonstrated that susceptibility testing with pMiniF-1 adequately reflects their known substrate specificity profiles. This system provides a reliable method for assessing β-lactamase substrate specificity and will facilitate comparative studies on the functional diversity and evolution of emerging β-lactamase variants.

## INTRODUCTION

β-lactamases are enzymes that inactivate β-lactam antibiotics and represent the predominant mechanism of β-lactam resistance in Gram-negative bacteria (1, 2). These enzymes are classified based on their structural and functional characteristics (2). Even a single amino acid substitution in a β-lactamase can result in a distinct resistance phenotype, leading to the emergence of recognized variant enzymes (3, 4). The number of identified β-lactamases has surged, with more than 5,700 unique enzymes currently cataloged in the National Center for Biotechnology Information Reference Gene Catalog (5).

Although kinetic parameters are essential for understanding enzyme-substrate specificity, not all emerging β-lactamase variant-substrate combinations have been characterized. Accurate and reliable determination of β-lactamase kinetic parameters is typically limited to specialized laboratories staffed by expert personnel. Achieving >95% enzyme purity (3) requires extensive optimization, including selective ion-exchange and/or gel filtration chromatography tailored to the enzyme, as well as careful control of buffer composition, pH, and salt concentration. Furthermore, the exponential increase in enzyme-substrate combinations further complicates the acquisition of kinetic parameters.

The impact of β-lactamase substrate specificity on β-lactam resistance (β-lactamase-mediated resistance) has been evaluated using diverse methods, making cross-study comparisons difficult (4, 6–10). For example, β-lactam MICs are often measured for transformants harboring various plasmids with cloned β-lactamase genes, which exhibit different expression levels due to variations in plasmid copy number (PCN) and promoter strength (4, 11). Researchers commonly use commercial β-lactamase-free expression vectors to clone these genes for antimicrobial susceptibility testing (AST)-based β-lactamase-mediated phenotypic evaluation (3). However, these vectors rely on inducible promoters, require antibiotic selection to maintain plasmids, and generally lack partitioning systems, resulting in unstable PCN (12–14). The copy number of plasmids carrying β-lactamase genes or duplication of β-lactamase genes influences the increase in β-lactam MICs through enhanced expression of β-lactamase in the host bacterium (15–18). For example, *Escherichia coli* isolates from one patient showed a >16-fold increase in cefiderocol MIC when the *bla*_NDM-5_ copy number increased from one to five by gene duplication (17). Thus, using a vector that is consistently maintained would enable more accurate comparisons of β-lactamase-mediated resistance by AST (19).

We selected the mini F plasmid, a truncated IncF replicon with intrinsic plasmid stability, which is reported to be maintained at one to two copies per cell (20–24), for comparing β-lactamase substrate specificity through AST. A derivative of the mini F plasmid previously served as a cloning vector, containing a T7 promoter within a multiple cloning site to transcribe cloned genes (25). In this study, to enable expression of genes cloned into the mini F plasmid and phenotypic evaluation by the broth microdilution (BMD) method, we modified the mini F plasmid pXX563 to construct pMiniF-1 plasmid by introducing a *tac* promoter (P*_tac_*) (20, 25, 26). This promoter allowed downstream gene expression without the use of inducers such as isopropyl-β-D-thiogalactopyranoside.

## MATERIALS AND METHODS

### Whole-genome sequencing

To confirm the complete sequence of pXX563 before constructing its derivative, pMiniF-1, whole-genome sequencing (WGS) was performed using *E. coli* carrying pXX563. After obtaining pMiniF-1 and β-lactamase gene cloned into pMiniF-1, transformants carrying them were also subjected to WGS to confirm that they had the expected genome structure. Genomic DNA was extracted from bacteria cultured on drug-free Mueller Hinton Agar (Becton Dickinson and Company, NJ, USA) using the MagDEA Dx SV PS protocol on the magLEAD 6gC (Precision System Science Co., Ltd., Matsudo, Japan). DNA libraries for MiSeq (Illumina, Inc., CA, USA) libraries were prepared using Illumina DNA Prep, (M) Tagmentation Kit (Illumina) and sequenced with 300 bp paired-end reads; reads were adapter-trimmed and quality-filtered (<Q30, length <100 bp) using Trimmomatic version 0.39. DNA libraries for MinION (Oxford Nanopore Technologies: ONT, Oxford, UK) were prepared using the Rapid Barcoding Kit 24 V14 (ONT) and run on MinION Flow Cell R10.4.1 (ONT); basecalling and demultiplexing were done in Dorado v0.9.0 (ONT). MinION reads were adapter-trimmed, filtered (<Q10, length <1,000 bp), and self-error corrected using DeChat (27). Hybrid *de novo* assemblies were generated with Hybracter version 0.11.2 (28). Gene annotation and antimicrobial resistance gene identification utilized the DNA Data Bank of Japan Fast Annotation and Submission Tool (29) and ResFinder (30). Chromosome and plasmid structures were visualized using Easyfig (31).

### Disruption of the chromosomal AmpC gene (*bla*_EC-like_) in *E. coli* DH5α

To prevent unwanted AmpC expression in *E. coli* DH5α, the chromosomal AmpC gene (*bla*_EC-like_) was disrupted using the one-step inactivation method (32). This PCR-based gene replacement employs a temperature-sensitive helper plasmid and leaves no antimicrobial resistance marker (Fig. S1). Detailed methods are in the Supplemental methods. Loss of *bla*_EC-like_ was confirmed by PCR and subsequent WGS.

### Modification of pXX563

**Fig 1:**
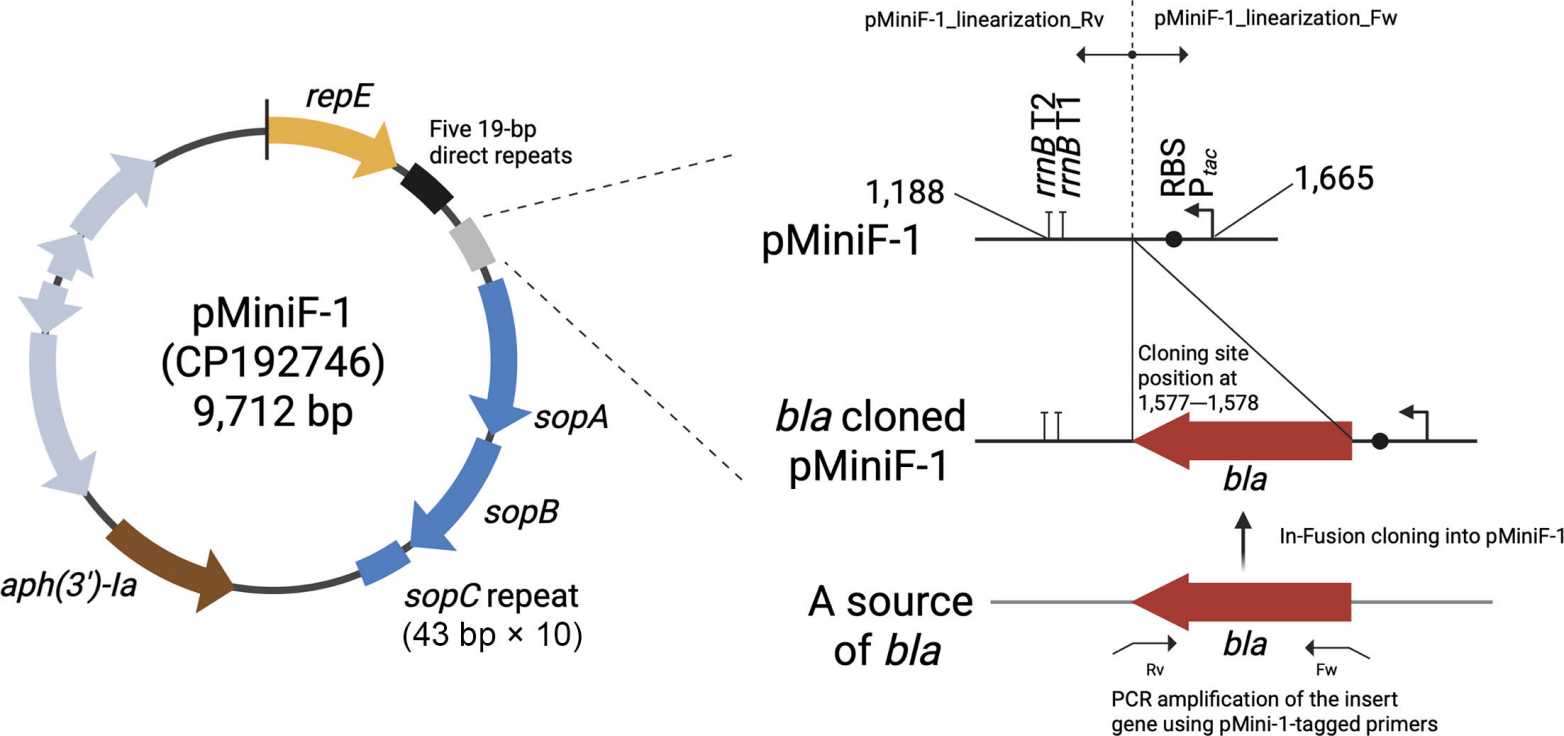
Structure of pMiniF-1. The insert gene is integrated between nucleotide positions 1,577 and 1,578 of pMiniF-1. P*tac*, *tac* promoter; *rrnB* T1/T2, ribosomal RNA gene terminators; RBS, ribosome-binding site. This figure was created with BioRender.com.

**Fig 2:**
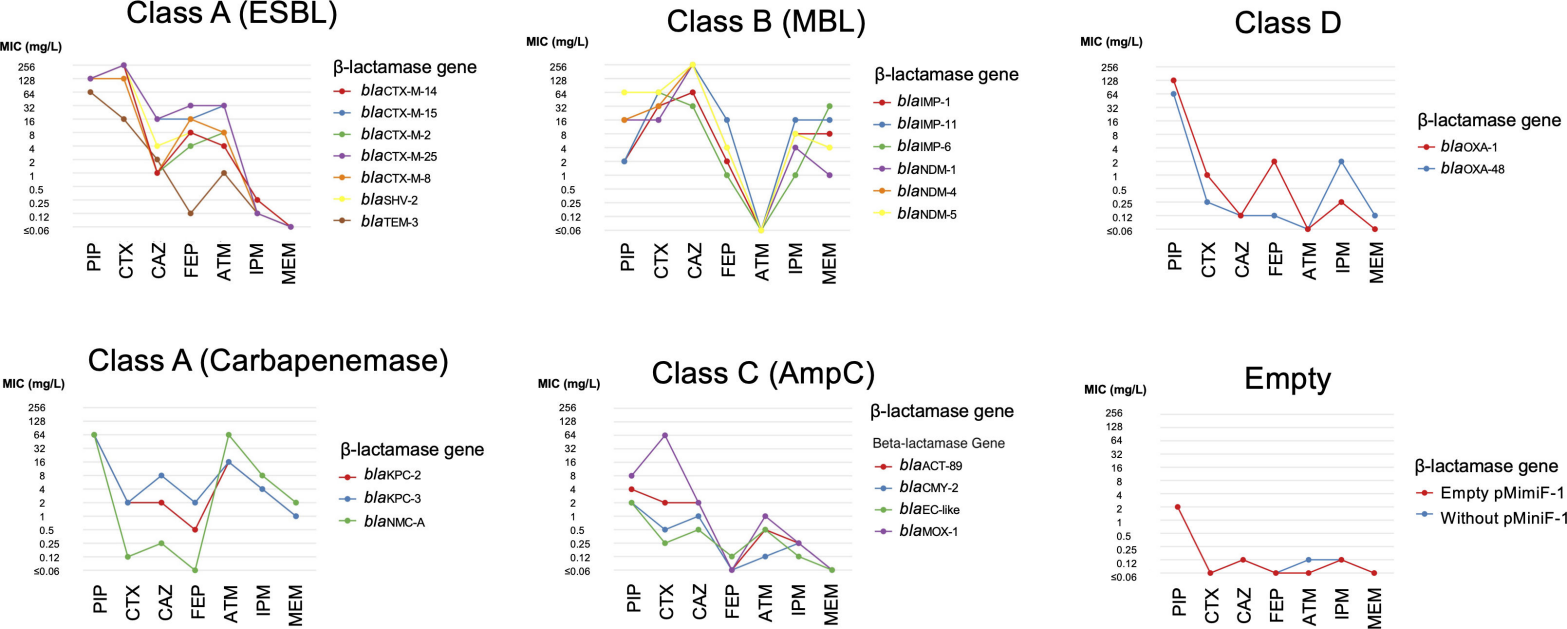
Antimicrobial susceptibility profiles of *E. coli* DH5αΔ*bla*_EC-like_ transformants harboring pMiniF-1 with cloned β-lactamase genes. Tested ranges were piperacillin (PIP, 2–64 mg/L), cefotaxime (CTX, 0.06–128 mg/L), ceftazidime (CAZ, 0.06–128 mg/L), cefepime (FEP, 0.06–128 mg/L), aztreonam (ATM, 0.06–128 mg/L), imipenem (IPM, 0.06–128 mg/L), and meropenem (MEM, 0.06–128 mg/L).

The mini F plasmid, pXX563, which natively lacks a multiple cloning site, promoters, terminators, and a ribosome binding site (RBS), was modified to express an inserted gene, resulting in the construction of the pMiniF-1 plasmid (32, 33). The *tac* promoter was employed to drive β-lactamase expression at levels that fall within the typical measurement range of the BMD method in clinical microbiology (26, 33). P*_tac_*, *rrnB* T1 and T2 terminators (*rrnB*-T1/T2), and RBS were integrated into pXX563 using in-fusion cloning technique (Fig. 1 and 2) (34). The *rrnB*-T1/T2 terminators and RBS were PCR-amplified from DH5α genomic DNA (Fig. S2). The P*_tac_* fragment was obtained by tail PCR with primers encoding the promoter sequence at their 5′ end (see Table S2 for primer sequences and PCR conditions). The pMiniF-1 plasmid has been deposited at the RIKEN BioResource Research Center (Tsukuba, Ibaraki, Japan) and is available under Catalog No. RDB21122 (35).

### Cloning β-lactamase genes into pMiniF-1

A total of 22 representative β-lactamase genes (Table 1; Table S1) were selected and cloned into pMiniF-1 using In-Fusion Snap Assembly Master Mix (TaKaRa Bio, Shiga, Japan). Primers were designed with TaKaRa’s online tools (https://www.takarabio.com/learning-centers/cloning/primer-design-and-other-tools, accessed September 2025) and are listed in Table S3. Inserts were integrated between P*_tac_*/RBS and the *rrnB* T1/T2 terminators at nucleotide position 1,577–1,578 of pMiniF-1 (Fig. 1). To guarantee efficient translation termination, the primers were designed to introduce a UAA stop codon (36). PCR was performed with PrimeSTAR GXL Premix Fast (TaKaRa Bio) following the conditions in Table S3. Competent *E. coli* DH5αΔ*bla*_EC-like_ cells were prepared and transformed by heat shock as described (37, 38). The transformants were stored at −80 °C in Mueller Hinton broth (MHB, Becton Dickinson and Company, NJ, USA) with 20% glycerol (FUJIFILM Wako Chemicals, Osaka, Japan) until use.

**TABLE 1:** Antimicrobial susceptibility with transformants by pMiniF-1 cloning β-lactamase genes^*a*^

β-Lactamase category	Strain ID	Host strain	β-Lactamase gene cloned into pMiniF-1	PIP	CTX	CAZ	FEP	ATM	IPM	MEM
Without β-lactamase	TUM22153	*E. coli* DH5α	pMiniF-1 negative	≤2	≤0.06	0.12	≤0.06	0.12	0.12	≤0.06
	TUM22199	*E. coli* DH5αΔ*bla*_EC-like_	pMiniF-1 negative	≤2	≤0.06	0.12	≤0.06	≤0.06	0.12	≤0.06
	TUM22775	*E. coli* DH5αΔ*bla*_EC-like_	Empty	≤2	≤0.06	≤0.06	≤0.06	≤0.06	0.12	≤0.06
Class A, ESBL	TUM23332	*E. coli* DH5αΔ*bla*_EC-like_	*bla*_TEM-3_	64	16	2	0.12	1	0.12	≤0.06
	TUM23331	*E. coli* DH5αΔ*bla*_EC-like_	*bla*_SHV-2_	>64	>128	4	8	4	0.25	≤0.06
	TUM23335	*E. coli* DH5αΔ*bla*_EC-like_	*bla*_CTX-M-15_	>64	>128	16	16	32	0.12	≤0.06
	TUM23337	*E. coli* DH5αΔ*bla*_EC-like_	*bla*_CTX-M-2_	>64	>128	1	4	8	0.12	≤0.06
	TUM23339	*E. coli* DH5αΔ*bla*_EC-like_	*bla*_CTX-M-8_	>64	128	1	16	8	0.12	≤0.06
	TUM23341	*E. coli* DH5αΔ*bla*_EC-like_	*bla*_CTX-M-14_	>64	>128	1	8	4	0.25	≤0.06
	TUM24769	*E. coli* DH5αΔ*bla*_EC-like_	*bla*_CTX-M-25_	>64	>128	16	32	32	0.12	≤0.06
Class A, carbapenemase	TUM22766	*E. coli* DH5αΔ*bla*_EC-like_	*bla*_KPC-2_	64	2	2	0.5	16	4	1
	TUM22767	*E. coli* DH5αΔ*bla*_EC-like_	*bla*_KPC-3_	64	2	8	2	16	4	1
	TUM22768	*E. coli* DH5αΔ*bla*_EC-like_	*bla*_NMC-A_	64	0.12	0.25	≤0.06	64	8	2
Class B, metallo-β-lactamase	TUM22760	*E. coli* DH5αΔ*bla*_EC-like_	*bla*_IMP-1_	≤2	32	64	2	≤0.06	8	8
	TUM22761	*E. coli* DH5αΔ*bla*_EC-like_	*bla*_IMP-6_	≤2	64	32	1	≤0.06	1	32
	TUM22762	*E. coli* DH5αΔ*bla*_EC-like_	*bla*_IMP-11_	≤2	64	>128	16	≤0.06	16	16
	TUM22763	*E. coli* DH5αΔ*bla*_EC-like_	*bla*_NDM-1_	16	16	>128	4	≤0.06	4	1
	TUM22764	*E. coli* DH5αΔ*bla*_EC-like_	*bla*_NDM-4_	16	32	>128	4	≤0.06	8	4
	TUM22765	*E. coli* DH5αΔ*bla*_EC-like_	*bla*_NDM-5_	64	64	>128	4	≤0.06	8	4
Class C, β-lactamase (AmpC)	TUM24563	*E. coli* DH5αΔ*bla*_EC-like_	*bla*_ACT-89_	4	2	2	≤0.06	0.5	0.25	≤0.06
	TUM24564	*E. coli* DH5αΔ*bla*_EC-like_	*bla*_PDC-1_	4	2	1	0.25	0.5	0.12	≤0.06
	TUM24566	*E. coli* DH5αΔ*bla*_EC-like_	*bla*_CMY-2_	≤2	0.5	1	≤0.06	0.12	0.25	≤0.06
	TUM24567	*E. coli* DH5αΔ*bla*_EC-like_	*bla*_MOX-1_	8	64	2	≤0.06	1	0.25	≤0.06
	TUM24570	*E. coli* DH5αΔ*bla*_EC-like_	*bla*_EC-like_	≤2	0.25	0.5	0.12	0.5	0.12	≤0.06
Class D, narrow-spectrum β-lactamases	TUM24562	*E. coli* DH5αΔ*bla*_EC-like_	*bla*_OXA-1_	>64	1	0.12	2	≤0.06	0.25	≤0.06
Class D, carbapenemase	TUM22769	*E. coli* DH5αΔ*bla*_EC-like_	*bla*_OXA-48_	64	0.25	0.12	0.12	≤0.06	2	0.12

^*a*^ ESBL, extended-spectrum β-lactamase; PIP, piperacillin; CTX, cefotaxime; CAZ, ceftazidime; FEP, cefepime; ATM, aztreonam; IPM, imipenem; MEM, meropenem.

### Antimicrobial susceptibility testing

A 96-well broth microdilution (BMD) panel (DTB1, Eiken Chemical Co., Ltd., Tokyo, Japan) was created to compare β-lactamase-mediated resistance phenotypes in pMiniF-1 transformants. Tested antimicrobials and ranges were piperacillin (PIP, 2–64 mg/L), cefotaxime (CTX, 0.06–128 mg/L), ceftazidime (CAZ, 0.06–128 mg/L), cefepime (FEP, 0.06–128 mg/L), aztreonam (ATM, 0.06–128 mg/L), imipenem (IPM, 0.06–128 mg/L), and meropenem (MEM, 0.06–128 mg/L) (Fig. 2). The measurement range of MICs is suitable to compare β-lactamase-mediated resistance under P*_tac_*-driven β-lactamase expression. AST was performed according to CLSI M07 guidelines (39): bacterial suspensions were adjusted to 0.5 McFarland in saline using a BD PhoenixSpec Nephelometer (BD), calibrated with the BD PhoenixSpec Calibrator Kit (BD), diluted 1:10, and 5 µL inoculated per well. Plates were incubated at 35°C for 18 h. *E. coli* ATCC 25922 and *P. aeruginosa* ATCC 27853 were used as quality controls.

### Phenotypic stability of pMiniF-1-*bla* transformants

To evaluate stability of the phenotypic characteristics in pMiniF-1-*bla* transformants using MEM MIC as an indicator, we used representative pMiniF-1 transformants carrying the one gene from each of the four major carbapenemase types (*bla*_IMP-6_, *bla*_KPC-3_, *bla*_NDM-5_, and *bla*_OXA-48_). Approximately 1.5 µL of a stationary-phase bacterial culture was inoculated into antibiotic-free Cation-Adjusted Mueller Hinton II Broth (BD) and incubated overnight at 35°C. This subculturing step was performed for a total of seven passages, more than the standard American Type Culture Collection protocol recommends using strains within five passages (40). After the final passage, the culture was supplemented with glycerol (FUJIFILM Wako Chemicals) to a final concentration of 20% and stored at –80°C until MIC testing. To assess the impact of serial passages in antibiotic-free media, MEM MICs were measured and compared between strains before and after passaging using DTML frozen BMD plates (Eiken) containing MEM at concentrations ranging from 0.015 to 16 mg/L, following the same procedure described above.

### Plasmid copy number

Total DNA was extracted using QIAamp DNA mini kit (QIAGEN, Hilden, Germany) from *E. coli* DH5αΔ*bla*_EC-like_ strains harboring either pMiniF-1 or pMiniF-1-*bla*, which had been grown to the stationary phase. The relative PCN of pMiniF-1 was determined via SYBR Green-based quantitative PCR (qPCR) targeting *aph(3′)-Ia* on pMiniF-1 and *dxs* on the chromosome (Fig. S3). *dxs*, present as a single copy in the *E. coli* genome, was used as an endogenous reference (41). Primer sequences and qPCR conditions are detailed in the Supplemental methods. The relative pMiniF-1 copy number was calculated using the ∆Ct method. All experiments were performed in biological triplicate. A transformant harboring the pHSG298 plasmid served as a high-copy-number reference (42). Data visualization was performed using the ggplot2 package in R version 4.4.2 (43).

### Quantification of transcripts of cloned β-lactamase genes

Total RNA was extracted from *E. coli* DH5αΔ*bla*_EC-like_ harboring either pMiniF-1 or pMiniF-1-*bla*, grown in MHB to the logarithmic phase (OD_600_ = 0.5–0.8), using the RNeasy Mini Kit (QIAGEN). To quantify transcripts of the cloned β-lactamase genes on pMiniF-1 in a cost-effective manner, we developed a targeted complementary DNA (cDNA)-sequencing method. Briefly, the transcripts of both the cloned β-lactamase genes on pMiniF-1 and the chromosomal *dxs* gene were simultaneously amplified via multiplex one-step reverse transcription-PCR (RT-PCR) with concurrent inner barcoding, based on a previously reported method (44) (Fig. S4). The primer sequences and one-step RT-PCR conditions are detailed in the Supplemental methods. The resulting amplicons were used to prepare sequencing libraries using the Native Barcoding Kit 24 V14 (ONT). Sequencing was performed on a MinION device equipped with R10.4.1 Flow Cells. The relative expression levels of the cloned β-lactamase genes were normalized to those of *dxs* using Transcript Per Million values. All experiments were performed in biological triplicate. The plot was generated using the R package ggplot2. Statistical significance was evaluated at the 0.05 level using Dunnett’s test, implemented in the R package multcomp, with the CTX-M-14 sample serving as the reference since it exhibited the median expression level among all tested samples (45).

## RESULTS

### Whole-genome sequence analysis of pMiniF-1-*bla* transformants

The structure of pMiniF-1 is shown in Fig. 1. We successfully cloned 22 different β-lactamase genes into pMiniF-1 (pMiniF-1-*bla*) and transformed *E. coli* DH5αΔ*bla*_EC-like_ with these constructs (Table 1). WGS of the transformants, including those carrying pMiniF-1 and pMiniF-1-*bla*, confirmed that (i) no unintended nucleotide changes occurred within the insert sequences on pMiniF-1-*bla*, (ii) pMiniF-1 retains an identical sequence to the copy number control systems of pXX563, including five 19-bp direct repeats downstream of *repE* and 11 tandem repeats of a 43-bp segment in *sopC* region (Fig. S2). These iterons and *sopC* repeats are essential components of the copy number control and active partitioning systems, respectively, that ensure stable, low-copy maintenance of mini F-derived plasmids (20, 22, 46), and (iii) the chromosomal AmpC gene in *E. coli* DH5α was successfully disrupted as expected.

### Antimicrobial susceptibility profiles of pMiniF-1-*bla* transformants

MICs for seven β-lactams were determined for transformants carrying pMiniF-1 and each pMiniF-1-*bla* plasmid (Table 1; Fig. 2). The following summarizes changes in MIC values of pMiniF-1-*bla* transformants compared to the pMiniF-1 control. The empty pMiniF-1 transformant served as a vector control, while the host strain without a plasmid was included to evaluate baseline susceptibility.

### Class A ESBLs

Compared with the pMiniF-1 control, transformants carrying class A extended-spectrum β-lactamase (ESBL) genes exhibited ≥16-fold increases in MICs of PIP, CTX, CAZ, and ATM. The MICs of FEP also increased by ≥16-fold, except for the TEM-3 producing transformant. In contrast, the MIC of IPM and MEM increased by ≤2-fold. Among the CTX-M producers, transformants expressing CTX-M-15 and CTX-M-25 demonstrated ≥256-fold increases in CAZ MICs, whereas the CTX-M-2, CTX-M-8, and CTX-M-14 producers showed increases of ≥64-fold. Notably, a 16-fold difference in the CAZ MICs was observed between the CTX-M-14 and CTX-M-15 producers.

### Class A carbapenemases

Transformants producing KPC-2, KPC-3, and NMC-A exhibited ≥32-fold increases in the MICs of PIP. Additionally, the MICs of ATM, IPM, and MEM increased by ≥256-fold, ≥32-fold, and ≥16-fold, respectively, across these transformants. For CTX and CAZ, the MICs increased by ≥32-fold in the KPC-2- and KPC-3-producing transformants, whereas the NMC-A producer showed only a 2- to 4-fold increase. Similarly, the MICs of FEP increased by ≥8-fold in the KPC-2 and KPC-3 producers but remained unchanged in the NMC-A-producing transformant.

### Class B β-lactamases

Transformants expressing class B β-lactamases exhibited ≥16-fold increases in the MICs for CTX, CAZ, IPM, and MEM. Notably, the MICs of ATM remained unchanged across all class B β-lactamase producers. Additionally, transformants producing IMP-1, IMP-6, and IMP-11 showed no increase in the MIC of PIP. Interestingly, an 8-fold difference in the IPM MIC was observed between the IMP-1- and IMP-6-producing transformants. Moreover, a substantial difference in the MIC of MEM was observed between the NDM-1 and IMP-6 producers (1 and 32 mg/L, respectively).

### Class C β-lactamases

Transformants producing class C β-lactamases exhibited ≥4-fold increases in the MICs of CTX and CAZ, whereas the MICs of FEP remained low. Among these transformants, the MICs of ATM increased by 2- to 16-fold. Notably, the MOX-1-producing transformant exhibited a substantially higher CTX MIC compared to other class C β-lactamase producers. Furthermore, the MIC of PIP increased markedly in this strain. The MICs of IPM and MEM remained unchanged.

### Class D β-lactamases

Transformants producing OXA-1 and OXA-48 exhibited ≥32-fold increases in the MICs of PIP. Notably, the OXA-1-producing transformant demonstrated 4-fold and 16-fold higher MICs of CTX and FEP, respectively, compared to the OXA-48 producer. In contrast, the OXA-48 producer showed an eightfold higher MIC of IPM and a slightly higher MIC of MEM compared to the OXA-1 producer.

### Stability of pMiniF-1-β-lactamase gene in host cells

Serial passage (seven daily transfers) of DH5αΔ*bla*_EC-like_ transformants carrying *bla*_IMP-6_, *bla*_KPC-3_, *bla*_NDM-5_, or *bla*_OXA-48_ on pMiniF-1 in antibiotic-free medium did not significantly alter MEM MICs (Table 2).

**TABLE 2:** Evaluation of pMiniF-1 stability in *E. coli* DH5αΔ*bla*_EC-like_ cells via measuring meropenem MIC

		Meropenem MIC (mg/L)						
		Days of serial passaging in antibiotic-free conditions						
Strain ID	Cloned gene into pMiniF-1	1	2	3	4	5	6	7
TUM22761	*bla*_IMP-6_	16	16	16	8	16	16	16
TUM22765	*bla*_NDM-5_	1	2	2	2	2	2	2
TUM22767	*bla*_KPC-3_	0.25	0.25	0.5	0.5	0.5	0.5	0.5
TUM22769	*bla*_OXA-48_	0.12	0.12	0.12	0.12	0.12	0.12	0.12

### Plasmid copy number

**Fig 3:**
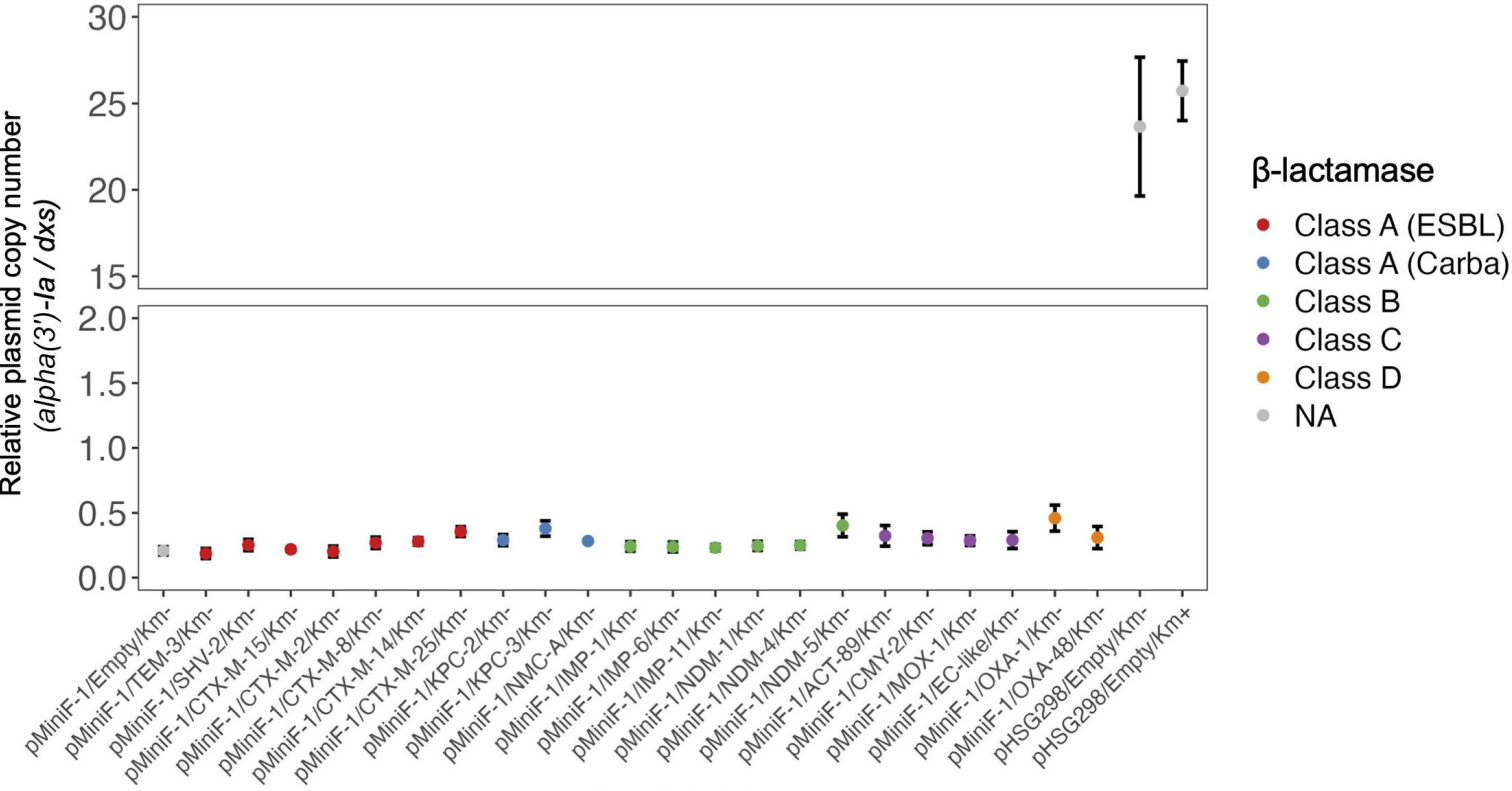
Relative Plasmid Copy Number determined by qPCR. The relative plasmid copy number was determined using the ∆Ct method based on qPCR data from biological triplicates. Data are presented as the mean, and error bars indicate the standard deviation (SD). Km+, cultured with kanamycin: 50 mg/L; Km−, cultured without kanamycin; NA, not applicable due to the absence of β-lactamase genes in the plasmid.

The relative PCN value of pMiniF-1, determined by qPCR, ranged from 0.19 to 0.46 per chromosome across 23 transformants by pMiniF-1 or pMiniF-1-*bla* during the stationary phase, with standard deviations (SDs) ranging from 0.01 to 0.10 (Fig. 3; Table S4). For pHSG298, the PCN values were 25.73 ± 1.72 and 23.65 ± 4.01 when cultured in the presence and absence of kanamycin, respectively.

### Transcript levels of cloned β-lactamase

**Fig 4:**
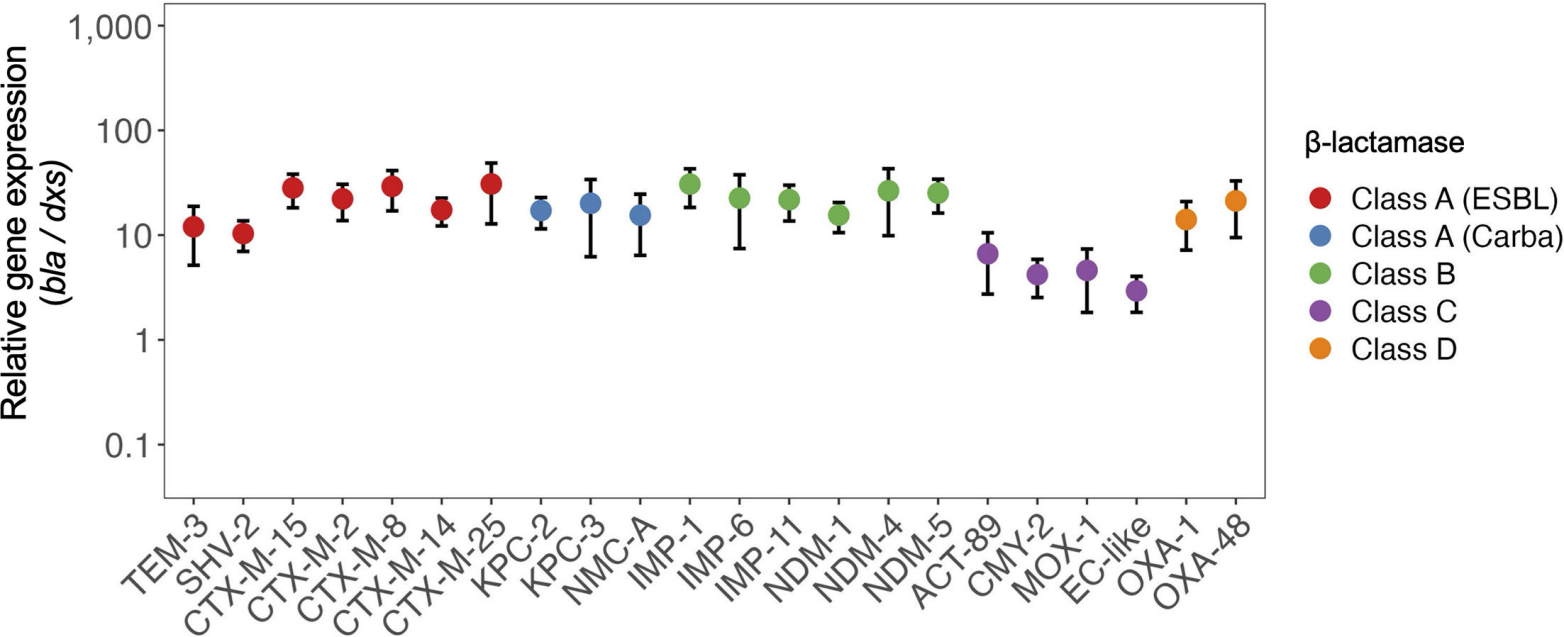
Relative transcription level of the cloned β-lactamase gene on pMiniF-1. The relative transcription level of the cloned β-lactamase gene on pMiniF-1 was measured using targeted cDNA sequencing from three biological replicates. Data are shown as the mean, with error bars indicating the standard deviation (SD). The relative expression level of the cloned β-lactamase genes was normalized to *dxs*.

Among the 22 pMiniF-1-*bla* transformants, the relative expression level of the cloned β-lactamase genes (normalized to *dxs*) ranged from 2.9 (pMiniF-1-*bla*_EC-like_) to 30.8 (pMiniF-1-*bla*_IMP-1_), with the highest variability observed in the CTX-M-25-producing transformant (SD = 18.0) (Fig. 4; Table S5). Despite this apparent variation, Dunnett’s test, using CTX-M-14-producing transformant as the reference due to its median expression value, revealed no statistically significant differences (*P* > 0.05) between the reference and any of the other transformants.

## DISCUSSION

The MICs for pMiniF-1 transformants producing various β-lactamase variants were largely consistent with previously reported kinetic parameters (Table 1; Tables S6 to S9). Specifically, the IMP-6 variant (Ser196→Gly) exhibited a fourfold increase in the MEM MIC but an eightfold decrease in the IPM MIC compared to IMP-1. The relatively low MEM MIC observed in the NDM-1 transformant may be attributable to differences in periplasmic enzyme accumulation, catalytic efficiency against MEM, or host-dependent factors affecting metallo-β-lactamase activity (47–50). Furthermore, the kinetic differences between OXA-1 and OXA-48 aligned well with their respective FEP MICs. In MOX-1-producing transformants, the observed PIP MICs also mirrored the expected trend derived from kinetic parameters, indicating a clear correlation between the two data sets.

However, comparing the compiled kinetic data with the MICs of pMiniF-1 transformants revealed several inconsistencies. For instance, although NMC-A exhibited substantially higher *k*_cat_/*K*_*m*_ values for CTX, IMP, and MEM compared to other β-lactamases, its corresponding MICs were not proportionally elevated. Similarly, while the CAZ *k*_cat_/*K*_*m*_ value of TEM-3 is approximately 100-fold higher than that of CTX-M-15, the CAZ MIC for the TEM-3-producing transformant was 8-fold lower than that of the CTX-M-15-producing transformant. These discrepancies underscore the need for caution when interpreting *k*_cat_/*K*_*m*_ values across studies.

Several factors may account for the discrepancies between the published *k*_cat_/*K*_*m*_ value and our MIC data. First, kinetic parameters determined *in vitro* using purified enzymes fail to capture *in vivo* variables, such as enzyme folding efficiency, periplasmic localization, protein stability, and effective substrate concentrations at the site of hydrolysis (51–55). Second, *k*_cat_/*K*_*m*_ values compiled from various studies often reflect variability in assay conditions (e.g., buffer composition, temperature, and substrate concentration ranges), which are rarely standardized across laboratories (56, 57). These considerations underscore the complementary value of whole-cell phenotypic assessment using a tightly controlled expression system such as pMiniF-1.

Although the basal expression of the chromosomal *bla*_EC-like_ gene in *E. coli* DH5α is negligible or absent, its promoter overlaps with the upstream *frd* operon, and the *bla*_EC-like_ attenuator functions as the terminator for *frd* transcription. This genomic organization implies that terminator instability or spontaneous mutations could theoretically trigger transcriptional readthrough, leading to subsequent *bla*_EC-like_ expression (3, 58). To rigorously exclude this possibility and ensure that the observed enzymatic activity is unambiguously attributed to the cloned gene, we used a knockout strain for transformation with pMiniF-1. Specifically, the MICs of CTX, CAZ, and ATM in the pMiniF-1-*bla*_EC-like_ strain (TUM24570) were ≥4-fold higher than those in the control strain (TUM22775) (Table 1). These findings indicate that verifying the absence of background *bla*_EC-like_ expression is essential for the accurate assessment of β-lactamase substrate specificity.

Determining the relative PCNs of pMiniF-1, pMiniF-1-*bla*, and pHSG298 by qPCR required adjustments for DNA topology. A previous study demonstrated that the relative PCN of a circular plasmid, when quantified by qPCR, appears to be approximately 20% of that of a linear plasmid (59). While small plasmids such as pMiniF-1 remain supercoiled and circular, the host chromosome carrying the reference gene (*dxs*) is inevitably fragmented during DNA extraction. Initially, the apparent relative PCN of pMiniF-1 determined by qPCR ranged from 0.19 to 0.46 copies per host chromosome. Because supercoiled circular plasmid DNA amplifies at roughly 20% of the efficiency of linearized chromosomal DNA during qPCR (59), dividing the observed value by this efficiency factor yields an estimated true copy number of approximately 1.0 to 2.3 per chromosome. This range aligns with the established maintenance of one to two copies per cell for the mini F plasmids regulated by the *repE*-interon and *sopABC* partition systems (20, 46). Furthermore, because the antimicrobial resistance phenotype conferred by pMiniF-1-*bla* remained stable for at least seven passages under drug-free conditions, these transformants can be reliably evaluated using standard AST in accordance with CLSI M07 guidelines (39).

Our analysis revealed that pHSG298, a widely used high-copy plasmid in *E. coli*, possessed a PCN approximately 100-fold higher than that of pMiniF-1. Notably, pHSG298 exhibited greater PCN variability in the absence of kanamycin selection pressure than when the antibiotic was present. Expressing β-lactamases from a high-copy plasmid can induce broad shifts in β-lactam MICs, generating inconsistent susceptibility profiles driven by fluctuations in PCN and plasmid stability. Consequently, these findings suggest that the pMiniF-1 system facilitates a more precise and reproducible evaluation of β-lactamase substrate specificities via AST than traditional high-copy expression methods.

The absence of statistically significant differences in *bla* transcript levels among the 22 pMiniF-1-*bla* transformants supports the premise that the *P*_tac_ drives comparable transcription levels regardless of the insert. However, the relatively broad range of mean expression levels (2.9–30.8 relative to *dxs*) and the high SD observed in certain transformants (up to 18.0) suggest that post-transcriptional factors—such as mRNA stability, insert sequence context, and translational efficiency—may contribute to the observed variability in MICs across different β-lactamase variants (60).

A limitation of the current system is its reliance on *E. coli* as the sole host organism. Because outer membrane permeability and efflux pump activities vary significantly among Gram-negative species, the MIC values obtained using *E. coli* transformants may not directly reflect the β-lactamase-mediated resistance levels observed in clinical pathogens such as *Pseudomonas aeruginosa* (61). Additionally, although NMC-A is an inducible chromosomal carbapenemase in the *Enterobacter cloacae* complex among Enterobacterales (62, 63), the pMiniF-1 system in *E. coli* does not recapitulate this inducible gene expression. Nevertheless, by employing a single, standardized host, the pMiniF-1 system eliminates host-to-host variability, thereby enabling a direct comparison of the intrinsic substrate preferences among diverse β-lactamases.

In conclusion, the pMiniF-1 system holds promise as a standardized platform for the rapid phenotypic characterization of newly identified β-lactamase variants, facilitating their functional classification alongside sequence-based identification. Furthermore, by pairing this system with a novel β-lactamase inhibitor to evaluate their efficacy across a comprehensive panel of enzymes under equivalent, single-copy expression conditions, it could help accelerate the preclinical evaluation pipeline.

## Data Availability

Sequence data have been deposited in GenBank under the BioProject accession number PRJNA1236865, and specific accession numbers are shown in Table S1.

## SUPPLEMENTAL MATERIAL

Supplemental material spectrum.03360-25-s0001.pdf Supplemental methods and Fig. S1 to S4.

Table S1 spectrum.03360-25-s0002.xlsx Information on pMiniF-1 and its transformants.

Table S2 spectrum.03360-25-s0003.xlsx Primers and PCR conditions used for the construction of pMiniF-1.

Table S3 spectrum.03360-25-s0004.xlsx Primers and PCR conditions used for cloning β-lactamase gene into pMiniF-1.

Table S4 spectrum.03360-25-s0005.xlsx Plasmid copy numbers of pMiniF-1, pMiniF-1-bla, and pHSG298 determined by qPCR.

Table S5 spectrum.03360-25-s0006.xlsx Expression level of the β-lactamase gene encoded by pMiniF-1, quantified by targeted transcriptome sequencing.

Table S6 spectrum.03360-25-s0007.xlsx Previously reported kinetic parameters of Class A β-lactamases used in this study.

Table S7 spectrum.03360-25-s0008.xlsx Previously reported kinetic parameters of Class B β-lactamases used in this study.

Table S8 spectrum.03360-25-s0009.xlsx Previously reported kinetic parameters of Class C β-lactamases used in this study.

Table S9 spectrum.03360-25-s0010.xlsx Previously reported kinetic parameters of Class D β-lactamases used in this study.

ASM does not own the copyrights to Supplemental Material that may be linked to, or accessed through, an article. The authors have granted ASM a non-exclusive, world-wide license to publish the Supplemental Material files. Please contact the corresponding author directly for reuse.
