# Supplementary material for: Development of a stable low-copy mini F plasmid derivative for evaluating β-lactamase substrate specificity through antimicrobial susceptibility testing
Source: Microbiol Spectr. 2026 Apr 16;14(6):e03360-25. doi: 10.1128/spectrum.03360-25 (PMC13228080; doi:10.1128/spectrum.03360-25)

Supplementary Methods

Disruption of the chromosomal AmpC gene (*bla*$_{EC\text{-}like}$) in *E. coli* DH5α

DH5α (F^-, Φ80d*lacZ*ΔM15, Δ(*lacZYA-argF*)U169, *deoR*, *recA1*, *endA1*, *hsdR*17(r_K^- m_K^+), *phoA*, *supE*44, λ^-, *thi*-1, *gyrA*96, *relA*1) was transformed with pKD46, which carries the phage λ Red recombinase genes under the control of an arabinose-inducible promoter(1). We then generated by PCR, using pKD4 as the template, a linear DNA cassette comprising (i) 50-nt homology arms flanking *bla*$_{EC\text{-}like}$, (ii) a kanamycin resistance gene, and (iii) an FLP recombinase target (FRT) site. The primers used were: KmR_H1_P1_DH5α: 5'-GTAAATCCGGCCCGCCTATGGCGGGCCGTTTTGTATGGAAACCAGACCCT gtgtaggctggagctgcttc -3', KmR_H2_P2_DH5α: 5'-CAGCAAGGAAAAGCGGAGAAAAGGTCCGAAAATTCGGACCCGATGGAATT catatgaatatcctcctta -3' (uppercase = genomic homology; lowercase = pKD4 sequence). PCR cycling conditions were as follows: 30 cycles of 98 ºC for 10 s, 55 ºC for 5 s, and 68 ºC for 10 s, using each 0.2 µM of each primer and PrimeSTAR GXL Premix Fast (Takara Bio Inc., Shiga, Japan).

The PCR product was purified after agarose gel electrophoresis and introduced into DH5α harboring pKD46 by electroporation. Recombinants were selected on agar plates containing 1 mM arabinose and 25 mg/L kanamycin at 30 °C. To cure pKD46, colonies were passaged on nonselective agar and incubated overnight at 37 °C. Next, to excise the kanamycin resistance marker, the cured strain was transformed with pCP20 (carrying the FLP recombinase gene and an ampicillin resistance gene), selected on agar containing 100 mg/L ampicillin at 30 °C, and pCP20 was subsequently cured by growth at 43 °C on nonselective agar.

Plasmid copy number analysis

The primer pairs used were dxs_Fw: 5'- ACACCCCGTTTGACCAATTG -3' and dxs_Rv: 5'- GAGGTTGATGAATGCCCGAC -3' for *dxs*, and aph_Fw: 5'-GGTTTGGTTGATGCGAGTGA -3' and aph_Rv: 5'-TTCCGACTCGTCCAACATCA -3' for *aph(3')-Ia*. qPCR was performed using the KAPA SYBR Fast qPCR Kit（ROX Low）(Kapa Biosystems, Inc., Wilmington, MA, USA) and a QuantStudio® 5 Real-Time PCR System (Thermo Fisher

Scientific/Applied Biosystems, Waltham, MA, USA). The qPCR conditions consisted of an initial denaturation at 95 ºC for 3 min, followed by 40 cycles of denaturation at 95 ºC for 3 s and annealing/extension at 60 ºC for 30 s. Melting curve analysis was performed using the instrument's default parameters. The linearity of the qPCR assay was verified using serially diluted DNA from *E. coli* harboring the pHSG298 plasmid (Figure S3).

Quantification of the cloned β-lactamase gene transcripts

Target gene transcripts were amplified by multiplex (*dxs* and *bla*) one-step reverse transcription-PCR (RT-PCR). The primer pairs used for the one-step RT-PCR were tailed with inner barcode (IBC) sequences(2). The primer sequences are listed in Table 1 of the Supplementary Methods. For each transformant, biological triplicates were analyzed using IBC1, IBC2, and IBC3. The multiplex one-step RT-PCR was performed using the PrimeScript One Step RT-PCR Kit Ver. 2 (TaKaRa Bio Inc., Shiga, Japan) under the following conditions: reverse transcription at 50 ºC for 30 min; initial denaturation at 94 ºC for 2 min; followed by 20 cycles of denaturation at 94 ºC for 30 s, annealing at 55 ºC for 30 s, and extension at 72 ºC for 90 s. The resulting PCR products were then pooled and purified using AMPure XP Beads (Beckman Coulter, Inc., Brea, CA, USA) prior to library preparation using the Native Barcoding Kit 24 V14 (Oxford Nanopore Technologies, Oxford, UK).

Table 1 in the Supplementary Methods. Primer sequences for the multiplex one-step RT-PCR

Target	Inner barcode	Forword (5'- -3')	Reverse (5'- -3')
dxs	1	GAGCCCGTTCCGCTCCATCAGTGCCGGAATTG	GAGCCCGTTCCGATTCGTTTTCATCGCTCGGG
	2	TGGCACCGATTACTCCATCAGTGCCGGAATTG	TGGCACCGATTAATTCGTTTTCATCGCTCGGG
	3	GACATACAATGACTCCATCAGTGCCGGAATTG	GACATACAATGAATTCGTTTTCATCGCTCGGG
bla	1	GAGCCCGTTCCGGCGGATAACAATTTCACACAGG	GAGCCCGTTCCGCCTACTCTCGCATGGGGA
	2	TGGCACCGATTAGCGGATAACAATTTCACACAGG	TGGCACCGATTACCTACTCTCGCATGGGGA
	3	GACATACAATGAGCGGATAACAATTTCACACAGG	GACATACAATGACCTACTCTCGCATGGGGA

The inner barcode sequence are underlined.

References:

1. Datsenko KA, Wanner BL. 2000. One-step inactivation of chromosomal genes in *Escherichia coli* K-12 using PCR products. Proc Natl Acad Sci U S A 97:6640–6645.

2. Dommann J, Kerbl-Knapp J, Albertos Torres D, Egli A, Keiser J, Schneeberger PHH. 2024. A novel barcoded nanopore sequencing workflow of high-quality, full-length bacterial 16S amplicons for taxonomic annotation of bacterial isolates and complex microbial communities. mSystems 9:e0085924.

Figure S1. Workflow for the disruption of the chromosomal AmpC gene (*bla*$_{EC\text{-}like}$) in *Escherichia coli* DH5α.
This figure was created with BioRender.com.

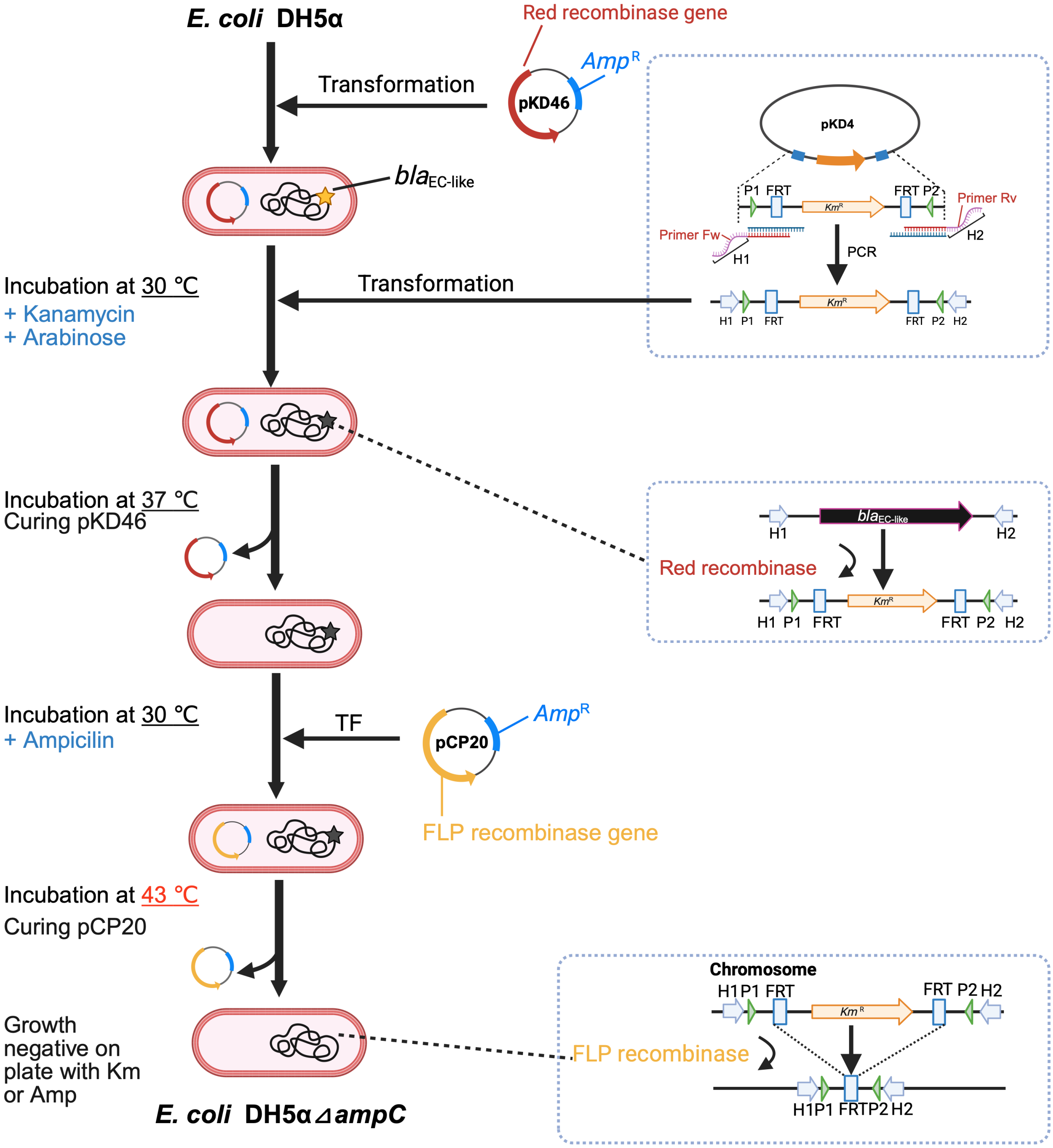

Figure S2. Workflow for the development of pMiniF-1.
This figure was created with BioRender.com.

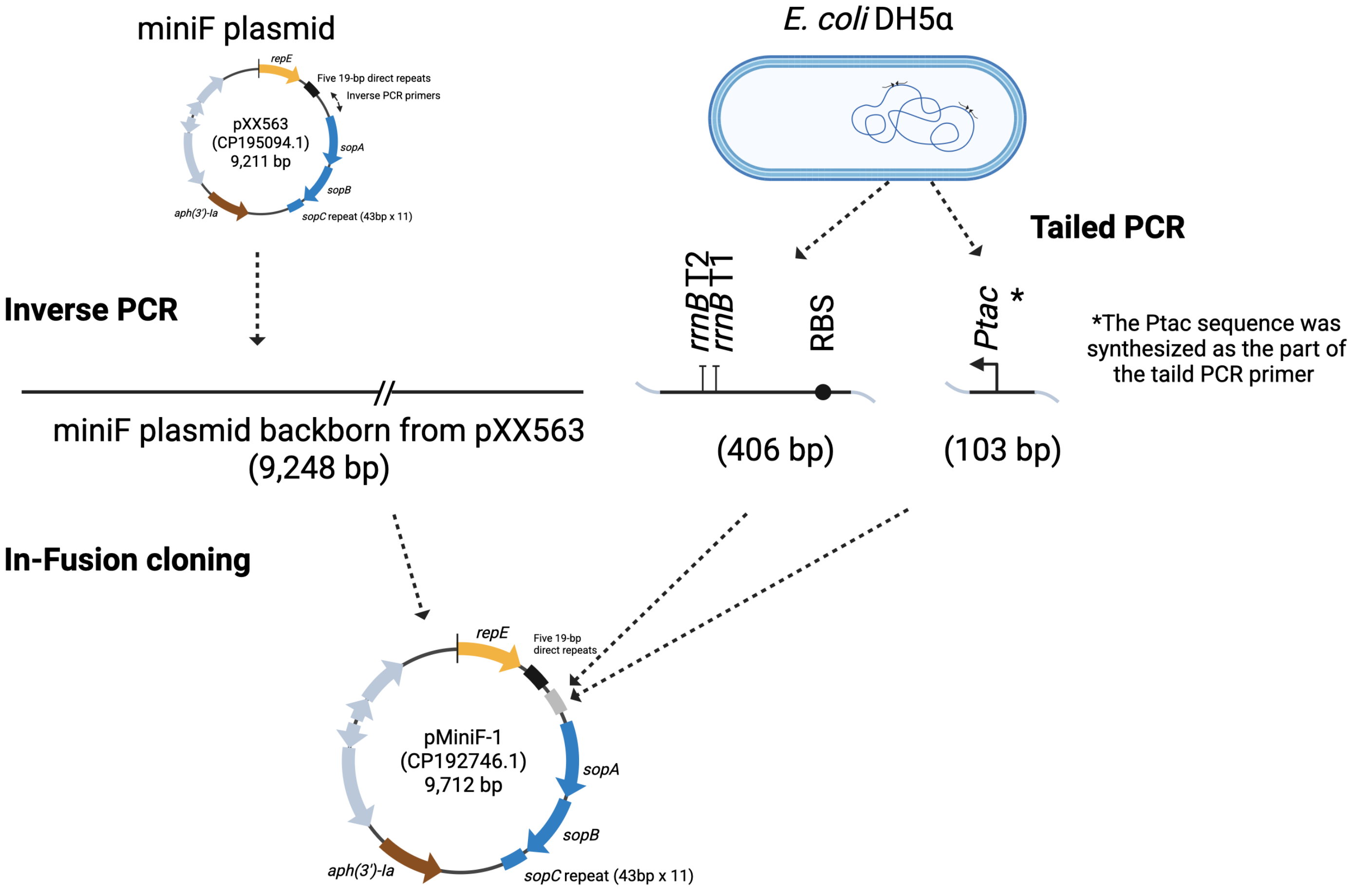

Figure S3. Determination of plasmid copy number by qPCR

A. Sample preparation and qPCR targets for relative plasmid Copy Number quantification
This figure was created with BioRender.com.

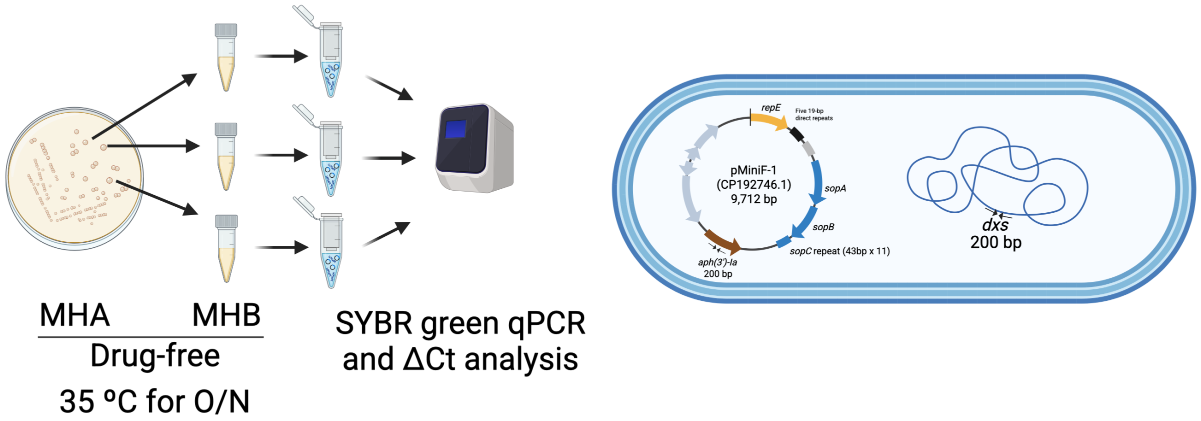

B. Linearity of the qPCR assay for relative plasmid copy number quantification

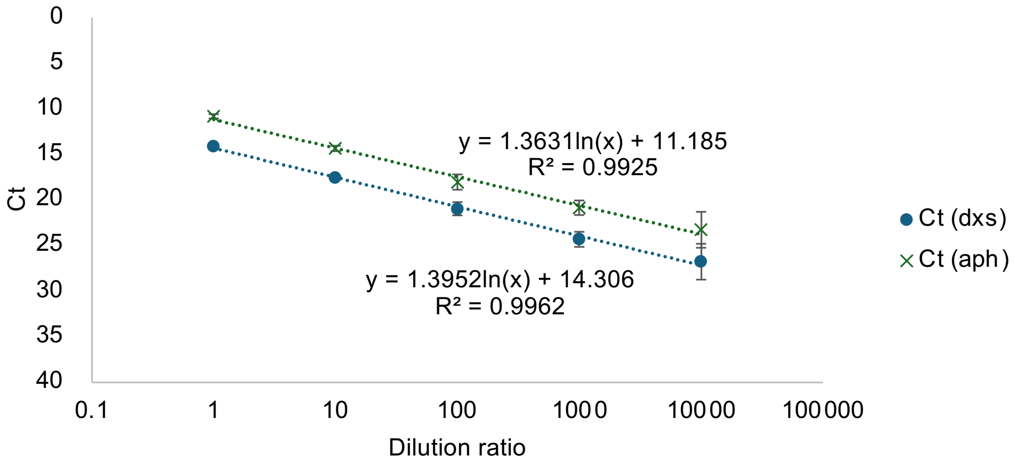

Figure S4. Workflow for the targeted transcriptome sequencing of cloned β-lactamase genes expressed from pMiniF-1

This figure was created with BioRender.com.

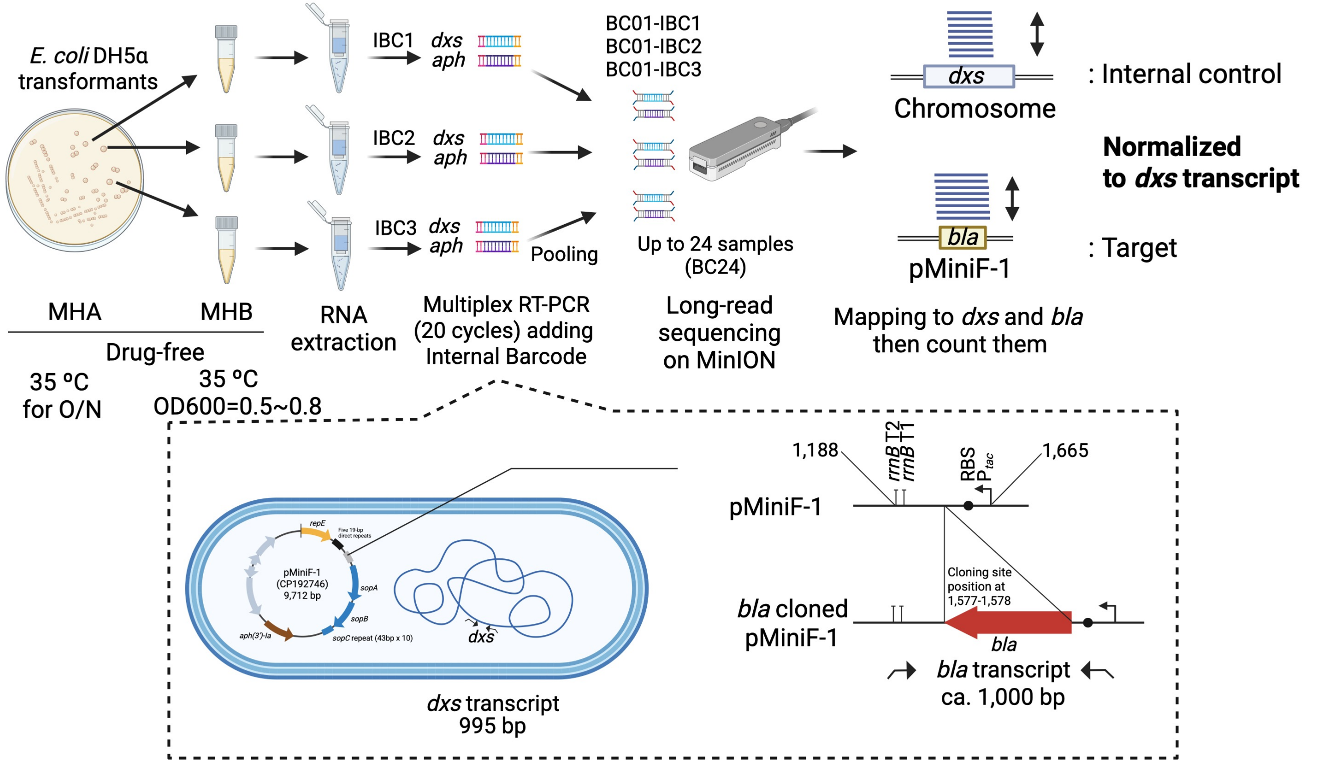

Supplement: Supplemental material — Supplemental methods and Fig. S1 to S4. [file spectrum.03360-25-s0001.pdf]
